# Causal evidence between monsoon and evolution of rhizomyine rodents

**DOI:** 10.1038/srep09008

**Published:** 2015-03-11

**Authors:** Raquel López-Antoñanzas, Fabien Knoll, Shiming Wan, Lawrence J. Flynn

**Affiliations:** 1 School of Earth Sciences, University of Bristol, Bristol, United Kingdom; 2 School of Earth, Atmospheric & Environmental Sciences, University of Manchester, Manchester, United Kingdom; 3 Departamento de Paleobiología, Museo Nacional de Ciencias Naturales-CSIC, Madrid, Spain; 4 Key Laboratory of Marine Geology and Environment, Institute of Oceanology-Chinese Academy of Science, Qingdao, China; 5 Department of Human Evolutionary Biology, Harvard University, Cambridge, USA

## Abstract

The modern Asian monsoonal systems are currently believed to have originated around the end of the Oligocene following a crucial step of uplift of the Tibetan-Himalayan highlands. Although monsoon possibly drove the evolution of many mammal lineages during the Neogene, no evidence thereof has been provided so far. We examined the evolutionary history of a clade of rodents, the Rhizomyinae, in conjunction with our current knowledge of monsoon fluctuations over time. The macroevolutionary dynamics of rhizomyines were analyzed within a well-constrained phylogenetic framework coupled with biogeographic and evolutionary rate studies. The evolutionary novelties developed by these rodents were surveyed in parallel with the fluctuations of the Indian monsoon so as to evaluate synchroneity and postulate causal relationships. We showed the existence of three drops in biodiversity during the evolution of rhizomyines, all of which reflected elevated extinction rates. Our results demonstrated linkage of monsoon variations with the evolution and biogeography of rhizomyines. Paradoxically, the evolution of rhizomyines was accelerated during the phases of weakening of the monsoons, not of strengthening, most probably because at those intervals forest habitats declined, which triggered extinction and progressive specialization toward a burrowing existence.

Ongoing climatic perturbation is a foremost concern for biodiversity preservation. Recent results suggest that global warming is likely to increase climatic variability and, therefore, impact the Asian monsoon^1^, which is one of the most widespread and mighty climatic phenomena on Earth^2^. The monsoon system comprises the East Asian and South Asian subsystems. The South Asian (or Indian) monsoon characterizes the climate south of the Himalayas, and throughout Indochina and the South China Sea, whereas the East Asian monsoon affects China and adjacent countries of eastern Asia^3^. The study of the Asian monsoon has attracted the interest of the scientific community due to its contribution to and influence on global climate variability, possibly as soon as its initiation around the Oligocene/Miocene boundary^4^. As a result, the evolution through time of monsoon systems and its correlation with the tectonic evolution of Asia are becoming better known. However, this does not hold true for our knowledge of the impact on resident faunas. Although drastic evolutionary responses may be expected for species living under monsoon-driven climatic conditions, no supporting data using phylogenetically constrained species-level information from the fossil record have been offered. By comparing rates of biodiversity change for a group of mammals studied by means of cladistic analyses, it is possible to calculate the rates of taxonomic turnover, which will allow inferences concerning the times of biodiversity crisis (net loss of biodiversity) and their causes (extinction or speciation decline)^5^. The growing knowledge of the fossil record and the growth of phylogenetic information coupled with biogeographic and evolutionary rate studies allow more detailed studies of the coevolution of the Earth and its biota^6^. Here, we unravel evolutionary rates and biogeographic patterns within rhizomyines, a group of mainly Asian subterranean rodents, and correlate the evolutionary history of these small mammals with fluctuations in monsoon strength since its origin in the Late Oligocene.

## Results

### Cladistic analysis

A single most parsimonious tree (Supplementary Figure 1) has been generated (see material and methods) with a length of 160, a Consistency index of 0.450, and a Retention index of 0.814.

### Speciation mode

The cladogram with biogeographic states mapped to terminal taxa and nodes (Supplementary Figure 1) shows that many of the transitions between nodes on the tree are not associated with major changes in geographic range. This indicates a predominance of differentiation within the biogeographic region over vicariant or geodispersal speciation in rhizomyine rodents. However, the evolutionary history of this group also involves episodes of vicariance and range expansion. In fact, since the Early Miocene the rhizomyines have experienced various dispersal events from Asia to Africa. The first would have taken place at approximately 19 Ma and would have concerned the basal clade of the rhizomyines, giving rise to the Libyan species *Prokanisamys* sp. During the Late Miocene, the rhizomyines would have experienced 6 unidirectional dispersal events (two from the Indian subcontinent to Africa and four from the Indian subcontinent to southeastern Asia). With respect to the intercontinental dispersals, the first would have given rise to the Ethiopian species “*Tachyoryctes*” *makooka*, whereas the second would have been at the origin of the African tribe Tachyoryctini (*Tachyoryctes* spp.). This tribe includes the single African living genus of the subfamily. As for the intra-continental dispersals, the first would have been at the origin of the tribe Rhizomyini, which encloses all Asian extant representatives of the subfamily (*Rhizomys* and *Cannomys*). The second and third dispersal events may not have taken place earlier than 9 Ma and concerned the taxa *Miorhizomys pilgrimi* and *M. tetracharax* plus *M. harii*, respectively. These species would have rapidly spread through southeastern Asia because similar forms have been recorded in southern China at about 8 Ma^7^. Finally, the last dispersal event would have given rise to the northern Chinese species “*Rhizomys* (*Brachyrhizomys*)” *shajius*.

**Figure 1:**
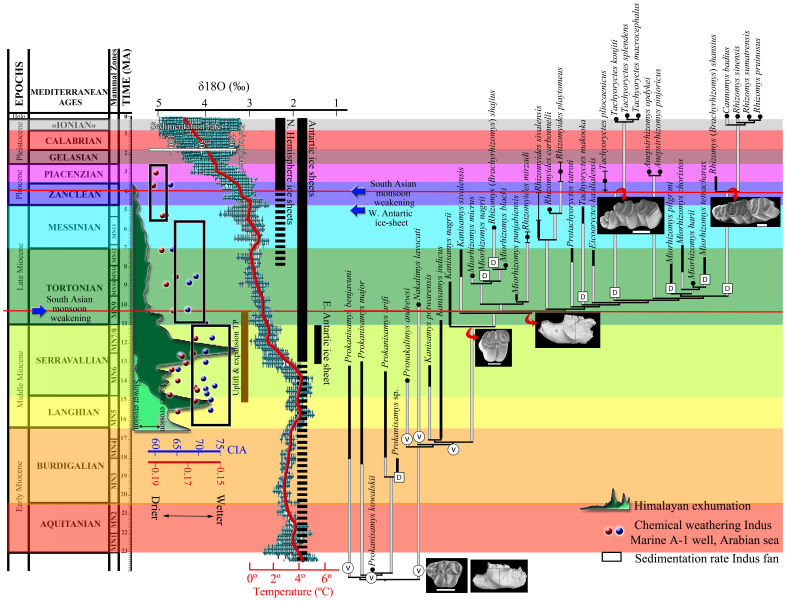
Calibrated phylogeny of rhizomyine rodents and their recorded temporal ranges (black). Grey bars represent missing ranges and missing ancestral lineages. Error bars indicate age uncertainty. Chronostratigraphical data from Sabatier^83^, De Bruijn et al.^84^, Flynn^28^,^51^,^63^,^68^, Flynn et al.^57^,^60^,^69^, Sen^85^, Wessels et al.^86^, Chaimanee et al.^87^, Wesselman et al.^88^, and Wessels^89^. Vicariance (V) and geodispersion (D) events are circled at the corresponding nodes. Red and blue dots correspond to K/Al ratios and Chemical Index of Alteration (CIA) data from cuttings from Indus Marine A-1 industrial well; black rectangles reflect the total sediment flux into the Indus fan; green surface to the probability densities for 40Ar/39Ar muscovite dates from the Himalayan hinterland (dark green) and proximal foreland (light green) (all taken from Clift et al.^11^). Global deep-sea oxygen isotope and temperature curves (red line) are from Zachos et al.^19^. Vertical bars provide approximation of ice volume in each hemisphere relative to the Last Glacial Maximum (LGM); dashed and full bars represent periods of minimal ice coverage (<50%) and maximal ice coverage (>50%), respectively. Blue arrows indicate periods of weakening of the South Asian summer monsoon. Red arrows point to the time of appearance of “key innovations” in rhizomyines (see text). Brown vertical bar indicates a phase of uplift and eastern expansion of the Tibetan Plateau.

The diversification of rhizomyines has also involved episodes of vicariance, particularly during the first two radiations. The first is near the origin of this group and the second matches the Early-Middle Miocene boundary (Fig. 1).

### Biodiversity analyses

**Figure 2:**
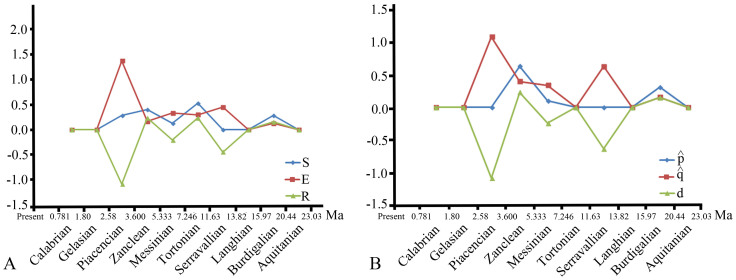
Instantaneous deterministic (A) and per-capita (B) rates for rhizomyine speciation, extinction, and biodiversity change calculated using phylogenetically constrained species ranges (see material and methods). Biodiversity rises in the Burdigalian, Tortonian, and Zanclean are driven by an increase in the speciation rates coupled with a lower extinction rate during the Tortonian. The three drops in biodiversity in the Serravallian, Messinian, and Zanclean are the result of elevated extinction rates.

Phylogenetically constrained per-capita rates (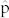, 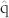, and d) and deterministic rates (R, S, and E) for the Rhizomyinae have been calculated (see material and methods). Results from the two sets of analyses have produced congruent patterns (Fig. 2).

Figure 2 shows that the biodiversity has changed during the course of the history of this group of rodents. The stages characterized by an increase in the biodiversity of the rhizomyines are the Burdigalian (20.44–15.97 Ma) and Tortonian (11.63–7.25 Ma) in the Early and Late Miocene, respectively, and the Zanclean (5.33–3.60 Ma) at the beginning of the Pliocene. The biodiversity of these rodents drops in the Serravallian (13.82–11.63 Ma, Middle Miocene), Messinian (7.25–5.33 Ma, Late Miocene), and Piazenzian (3.60–2.58 Ma, Pliocene). High speciation rates account for the increase in diversity during the Burdigalian and Zanclean, whereas the rise in biodiversity during the Tortonian is caused by elevated speciation rates coupled with low extinction. The three drops in biodiversity are the product of elevated extinction rates. The major biodiversity crisis endured by this group of rodents took place during the Pliocene, but a limited fossil record does not allow precise dating.

## Discussion

Much evidence suggests that climate changed both in and near India around 11–10 Ma, 8–7 Ma, and 4 Ma^2^,^8^,^9^,^10^,^11^,^12^,^13^. However, despite the fact that the Asian monsoon system has been dominating the Asian climate since its initiation close to the Oligocene/Miocene boundary^11^,^13^,^14^,^15^, no attempts have been made to infer its influence on specific mammal lineages. This is paradoxical because physical environmental factors are crucial in triggering evolution, particularly at large geographic and temporal scales^16^. There is a strong causal link among speciation, extinction, and environmental change, and one would expect to find pulses of faunal turnover linked to external factors such as climatic changes^17^. Combining the results of analyses of biodiversity and phylogenetic biogeography can reveal mechanisms responsible for the fluctuations of biodiversity through time.

### Early-Middle Miocene (23.03–11.63 Ma)

The timing of development of the South Asian monsoon is poorly constrained. Some authors cautiously infer its initiation around the Oligocene/Miocene boundary as proposed for the East Asian Monsoon^13^. Numerous works^11^,^15^ suggest a strong monsoon during the Early and Middle Miocene. Limited evidence indicates that southern Asian Early Miocene environments were moist and subtropical^18^. The late Early to early Middle Miocene, a period of warm global conditions^19^ (Fig. 1), would have favoured stronger summer monsoons^2^. Accordingly, the Middle Miocene faunas of Pakistan suggest moist conditions and the existence of a relatively closed rainforest^20^,^21^,^22^,^23^. Primitive rhizomyines (*Prokanisamys* spp.) show bunodont and rather brachydont cheek teeth that are consistent with the non-abrasive diet that is typical for moist habitats. Known osteological features of the skull and postcrania of these early rhizomyines do not support commitment to a fully fossorial lifestyle.

### Late Miocene (11.63–5.33 Ma)

During the early Late Miocene (~10 Ma), an important faunal turnover is observed in Siwalik rhizomyines (Fig. 1). The extinction of the primitive taxa with weak or moderate lophodonty (transverse crests as high as cusps) is concurrent with the diversification of the more derived, lophodont species of the group, which showed adaptations to an abrasive diet after 10.2 Ma (e.g., increase of the dentary depth, constriction of the mure in the cheek teeth, Fig. 1) and progressive adoption of fossorial features. This turnover is coincident with the end of the first biodiversity crisis in rhizomyines (Serravallien) (Fig. 2). In fact, the drop in the biodiversity of the group in the Serravallian (13.82–11.63 Ma) is due to the disappearance during this interval of nearly all the taxa that had crossed its lower boundary. This crisis is followed by a rapid and ample diversification of this group during the following interval, the Tortonian (11.63–7.25 Ma), that concerns mainly members of the fully subterranean lineages (Fig. 2). A more striking drop in biodiversity followed at the end of the Miocene, during the Messinian (7.25–5.33 Ma), when most of the lineages that originated and diversified during the Tortonian did not survive into the following stage. Late Miocene rhizomyine biodiversity mostly concerns representatives from the Indian subcontinent and southern Asia to the East, known today as the Oriental Biogeographic Province. Outside this area a single species is found in eastern Asia at the end of the Messinian^24^. Merely two taxa have been recorded from the Late Miocene of the whole African continent^25^,^26^.

The increase of lophodonty and hypsodonty in rhizomyines points to a diet of coarser vegetation and, therefore, suggests an increasing Asian climatic dryness around 10 Ma. At approximately 10.2 Ma, the dentary depth of rhizomyines increased from weak to moderate in all taxa. The deepening of the dentary together with the development of a heavy, flattened incisor is a good indicator of an adaptation to fossorial lifestyle in this group because living rhizomyines use mainly the incisors and not the limbs in burrowing^27^,^28^. In addition, at 9.8 Ma *Tachyoryctes makooka* and more derived taxa have the mure of the molars constricted. The beginning of mure loss can also be related to the adaptation to a fossorial way of life in rodents that dig their burrows mainly by the incisors, and consume subterranean plant structures. In such animals, the skull undergoes structural specialization and the same holds true for the dentition. Most subterranean rodents subsist on coarse food (e.g., roots, rhizomes, young twigs). Because simple crushing is no longer sufficient for these substances to be reduced, shearing and slicing dominate. Therefore, subterranean rodents feed by incision, followed by grinding with their molars, in contrast with arboreal forms, which generally eat by gnawing and then crushing^29^. Additionally, the rate of dental wear by attrition increases because the coarseness of food is greater and more grit is encountered in the subterranean diet. This is responsible for changes in dental pattern. The incisors become broader and chisel-shaped and the low crowned cuspidate molars become more hypsodont with flattened crown surfaces due to the submergence of the main tubercles into transverse folds^29^.

The beginning of the development of a subterranean way of life in open environments is advantageous for small mammals. The burrows provide shelter from predators, prevent excess water loss and permit controlled ambient temperature fluctuations. The triggering effect of open environments on fossoriality has been evidenced in caviomorph rodents^30^,^31^,^32^. For rhizomyines, the development of open environments in which food resources are reduced and the risk of predation is higher presumably drove primitive lineages to extinction and prompted the diversification of more derived ones with nascent adaptations to burrowing. The appearance of a subterranean lifestyle is, therefore, the chief factor that allowed rhizomyines to radiate after 11 Ma. The members of this radiation, the Tribe Rhizomyini, would have built underground burrow systems to avoid predators, and possibly would have begun to exploit underground plant structures.

Weakening of South Asian summer monsoon (weaker summer rains) and associated drying since the beginning of the Late Miocene would have shifted accordingly the habitat of rhizomyine rodents. The uplift and eastern expansion of the Tibetan Plateau over the past 15–10 Ma seem to have played a decisive role in the evolution of the South Asian monsoon^12^,^33^, which controls the climate of northwestern India and Pakistan. Since the beginning of the Late Miocene, climatic changes affected the Asian continent in general and the Indian subcontinent in particular, with increasing seasonality. An increase in seasonal aridity seems to be tied to the growth of eastern Tibet^33^. However, whether these climatic changes reflected a monsoonal strengthening (i.e., heavier summer rains) or, conversely, a weakening of monsoon rains is controversial. In fact, the formerly-accepted strengthening of the monsoon at this time is being increasingly questioned^11^,^13^,^33^,^34^,^35^,^36^,^37^. The benchmark works of Kroon et al.^8^ and Prell et al.^9^ on foraminifera and radiolarian assemblages in the western Arabian Sea have been particularly influential in this respect. The sharp increase in the abundances of *Globigerina bulloides* and *Actinomma* sp. at about 8–9 Ma indicates an intensification of upwelling strength in the Arabian Sea, which suggests monsoon strengthening at that time. Subsequent findings from the Indian subcontinent, including isotopic, faunal, and floral data have been assimilated into that view^10^,^23^,^38^,^39^,^40^. However, other works^41^ in the same area identified no marked increase in *G. bulloides* abundance at this time but important changes in carbon and hydrogen isotopic ratios of leaf waxes, which indicate an increase of the regional aridity. An emerging view that is more consistent with an increase in aridity over the northwestern Indian subcontinent is that the monsoon did not strengthen but weaken at this time. For example, Clift et al.^11^ identified slower erosion rates in the Himalayas since 10.5 Ma that correlated with a decrease in precipitation. This corresponds to a weakening of the Asian Summer Monsoon, possibly linked to a strong global cooling^19^ (Fig. 1). This interpretation was based on the observation that the regions of the Himalayas with the heaviest precipitation have maximal erosion rates today (see discussion in Clift & Plumb^2^). Consequently, Clift & Plumb^2^ interpreted the sharp drop in the rate of clastic accumulation in the Bengal fan and in some parts of the Himalayan foreland basin after 8 Ma found by Burbank et al.^42^ as a weakening of the monsoon. Furthermore, Chemical Index of Alteration values of sediments eroded from regions in which the South Asian monsoon dominates the climate, which represent chemical weathering in the western Himalayas over the period 17.0–3.0 Ma, have also generally been decreasing since the Middle Miocene, confirming the weakening trend of South Asian summer monsoon^11^,^13^ (see Fig. 1). Climate modelling results are perfectly consistent with a Late Miocene decrease in continental humidity^43^,^44^,^45^. A weakened South Asian summer monsoon would have limited the penetration of moisture from the ocean to the continent and, hence, increased the aridity in northwestern India and Pakistan, with far-reaching ecological and environmental shifts.

**Figure 3:**
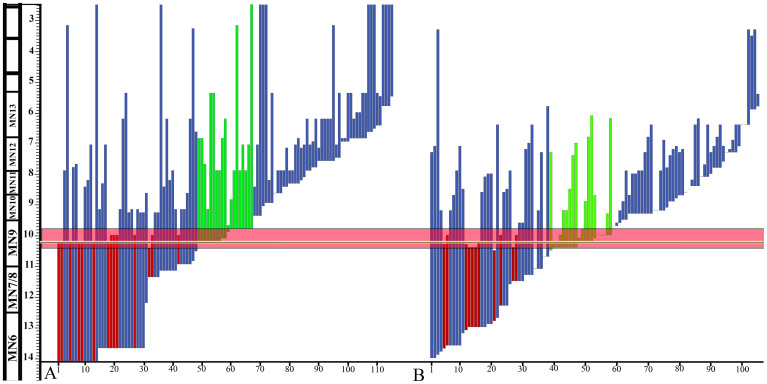
Biostratigraphic ranges of mammalian lineages from the Siwalik record (first and last occurrence inferred), late Miocene of the Potwar Plateau, after data from Barry et al.^46^ (A) and Badgley et al.^53^ (B). Species that went extinct between 10.4 and 9.8 Ma are in red, whereas appearances at that time are in green. The horizontal line in excess of 10 Ma indicates the initiation of the weakening of the monsoon, and coincides with the beginning of radiation of fossorial rhizomyine rodents.

There are numerous palaeontological studies that support an important climatic change in the Himalayan area since the beginning of the Late Miocene. For instance, after 12 Ma, the δ^18^O of fossil large mammal tooth enamel records a shift toward a drier environment in Pakistan^23^. In addition, the exceptional Siwalik fossil record has allowed the identification of a period of high faunal turnover at about 10.3 Ma that is characterized by a high level of disappearance^46^. In fact, more than 30% of the whole mammal fauna disappeared (11 out of 36 taxa) and 17 new taxa appeared (Fig. 3). Furthermore, the most significant faunal change in bovids occurs not long after 11 Ma^47^, nearly coinciding with the arrival of hipparionine horses. The rodent fossil record shows important changes such as the decline of cricetids, which had dominated the Siwalik Miocene since 18 Ma, and the local extinction of ctenodactylines^48^. Furthermore, Kimura et al.^49^ identified at this time (10.5 to 10.2 Ma) the first significant divergence in molar shape between the two murines *Karnimata* and *Progonomys*, which could be linked to environmental changes. Flynn^68^,^50^,^51^ observed an increase in hypsodonty in Late Miocene rhizomyines from the Pakistan Siwaliks. This has been tentatively correlated with a transition to a drier and more seasonal climate beginning around 9.2 Ma, which intensified by 7.8 Ma, as evidenced by changes in the floodplain deposition and vegetation^46^. The change in rodent fauna evidenced in the area by Flynn & Jacobs^52^ at about this time also supports an increase in aridity and a change in forest ecology.

A weakening of the South Asian summer monsoon would also explain the 7.3 to 7.0 Ma faunal turnover found by Barry et al.^46^ and Badgley et al.^53^. However, the decline of the C3-dominated vegetation (mostly trees and shrubs) and the dominance of plant communities with predominantly C4 vegetation (warm season grasses) around 7.4 Ma^46^, which has been attributed to monsoon intensification, is now believed to be rather due to the global cooling effects of a decrease in atmospheric CO_2_ at this time^54^. Similar changes have been identified in North America, far from the influence of the Asian monsoon^2^. Finally, the analyses carried out by Dettman et al.^10^ on isotopes of fossil bivalves and teeth from Nepal and Pakistan suggested a significant shift in climate toward a harsher aridity starting ca. 7.5 Ma.

### Pliocene (5.33–2.58 Ma)

The third and last biodiversity crisis experienced by the rhizomyines is recorded in the Piazencian (3.6–2.58 Ma). This is the major crisis endured by this group of rodents and is characterized by elevated extinction coupled with low speciation rates (Fig. 2). This drop in diversity occurred after a shift in climate toward drier conditions and a decrease of the South Asian summer monsoon at about 4–3 Ma, possibly linked to additional global cooling that reflects the onset of successive Northern Hemisphere Glaciations^19^ (see Fig. 1). This climatic change is supported by diverse results using different climatic proxies^11^,^14^,^55^. The closure of the Indonesian seaway by 4–3 Ma may have played a role in the intensification of Asian aridity, which would have resulted in not only a general decrease of the South Asian summer monsoon^14^ but also in an increase in the aridity of eastern Africa^56^. By 3 Ma, rhizomyines had suffered an important decline in the Indian subcontinent and surrounding areas, where they nearly disappeared. At that time, only two taxa survived in this area: the single bispecific genus *Anepsirhizomys* Flynn, 1982^68^ in Pakistan and India^68^,^57^ and *Rhizomyides* Bohlin, 1946^58^ in Afghanistan and northern India^59^,^60^. Both lineages show some significant adaptations in line with an increase in aridity. For instance, by 3.5 Ma the lineage of *Anepsirhizomys* developed hypsodont teeth and lost the mure on the second molars^68^. *Rhizomyides* *platytomeus* (3 Ma) also shows high crowned teeth, a dentary of moderate depth, and chisel-shaped incisors. Additional palaeontological data support the presence of open and arid or semiarid environments at this time in the region^61^. Increasing aridity possibly provoked the decline in biodiversity and local extinction of rhizomyines in the northern Indian subcontinent, where they would never return.

Paradoxically, while rhizomyines declined, the crown group survived north of its present range, dispersing into Shanxi, China. The Pliocene *Brachyrhizomys* lineage is preserved in deposits of the Yushe Basin, and the mid-Pliocene species *Rhizomys* (*Brachyrhizomys*) *shansius*^24^,^62^,^63^ is well known. By 4 Ma, this basal taxon of the crown Rhizomyini^64^, developed hypsodonty, the dentary depth and skull height increased from moderate to deep, and the ventral slit of the infraorbital foramen was eliminated by expansion of the masticatory musculature. All these characters point to an adaptation to a subterranean lifestyle, consistent with an increase in aridity.

From the Pliocene to the present, African rhizomyines have remained restricted to the single genus *Tachyoryctes* (*T. pliocaenicus* + more derived taxa), which shows discontinuous spatial distribution in the central and, especially, eastern parts of the continent. Recent studies in eastern Africa support the occurrence of an important shift in the vegetation at this time, consistent with an increase in aridity and seasonality and, therefore, an opening of the environment in this area by 3 Ma^65^,^66^.

Successive species of *Tachyoryctes* show an increase in hypsodonty through time^67^. Furthermore, the members of this clade share the synapomorphic loss of the mure on the molars by 4 Ma. As seen above, the loss of the mure and an increase in hypsodonty are consistent with the acquisition of burrowing habits, which were advantageous for these small mammals in open environments.

## Conclusion

Phylogenetic corrections of diversity estimates provide continuously improving paleontological data with a huge potential for understanding the macroevolutionary impact of environmental changes. A comprehensive cladistic analysis of rhizomyine rodents and subsequent estimates of speciation and extinction rates as well as diversity changes of the various lineages show that monsoon variations impacted the evolutionary history of this group of mammals. Thus, our data and analyses provide the first evidence of a correlation between monsoon variations and the evolution of a group of mammals in southern Asia. Counterintuitively, these are the phases of weakening of the monsoons (which began at about 10.5 Ma), not of strengthening, that have triggered the evolution in this part of the world in this specialized clade because they provoked a decline of forest habitats that precipitated their extinctions and progressive commitment toward a burrowing mode of life, which would have provided them with not only underground resources but also refuge from predators in an opening environment. The high rate of speciation of rhizomyines, particularly since the beginning of the Late Miocene, has been possibly exacerbated by isolating mechanisms correlated with a solitary lifestyle and small home ranges. The solitary way of life of the rhizomyines may have impacted the survival of these animals in an increasingly arid environment, provoking their dispersal and local extinction in much of South Asia during Pliocene time.

It is likely that on the scale of millions of years the monsoon system drove the evolution of many other taxa than rhizomyid rodents. Unfortunately, there is a limited phylogenetic framework to defend this for most other mammals. Hence, we are forced to rely only on a fossil record of uneven quality. The most thorough southern Asian paleobiological analyses, which have taken the fossil record at face value, are consistent with our results and offer evidence of some important faunal turnovers in the Siwaliks since the Late Miocene (particularly after 10.5 Ma). Many initiatives focus on climatic events to explain the faunal change from 8.5 Ma to 6 Ma, but the earlier important faunal turnover (at about 10.3 Ma), in which a third of the whole Siwalik mammal fauna disappeared and numerous new taxa appeared^46^, has been neglected. This event is synchronous with the most significant turnover that we found in rhizomyine rodents. Our hypotheses based on paleobiological analyses using phylogenetically constrained species-level information coupled with biogeographic and evolutionary rate studies offer a new perspective to conservation biologists working in Monsoon-dominated lands.

## Methods

Rodents are a choice study material because they are the most abundant mammals in the terrestrial fossil record, they generally show rapid evolution, and they are habitat-sensitive. This makes them the most informative group of mammals from a paleoenvironmental viewpoint. Amongst rodents, we chose to turn our attention to rhizomyines because (1) their phylogeny has been recently elucidated by means of cladistic analyses^64^, and (2) their evolutionary history took place mostly in southern Asia, where their fossil record is well known particularly thanks to the Miocene deposits of the Siwaliks^68^,^69^. Rhizomyines are, therefore, at one and the same time a group whose phylogenetic topology is well known, but whose driving evolutionary forces are unidentified even though the South Asian monsoon can be suspected *a priori* to have played a role.

### Cladistic analysis

The calculation of speciation rates and the assessment of speciation patterns require a detailed knowledge of the temporal framework and phylogenetic relationships of the considered taxa. This kind of information is supplied by cladograms calibrated in time (stratocladograms) in which the operational taxonomic units are species^5^. The evolutionary relationships of rhizomyine rodents have been studied in detail by López-Antoñanzas et al.^64^. The analyses of the evolutionary rates and paleobiogeography of rhizomyines that we present in this work build upon the character/taxon matrix from therein. As running that data matrix produced several trees^64^ and there is evidence of the improvement of phylogenetical analyses of morphological datasets when applying implied weighting^70^, we have downweighted characters according to their homoplasy. The matrix has been processed with the phylogenetic reconstruction software TNT^71^ under weak implied weighting with a concavity value of 8 (k = 8). Branch support was estimated through two complementary indices: Bremer Support^72^ and Relative Bremer Support^73^.

The resultant cladogram (Supplementary Figure 1) has been chronostratigraphically calibrated as a “stratocladogram” (Fig. 1), in which the chronostratigraphic range of any given taxon in the fossil record is completed backward to its origin according to the tree topology. Rhizomyine species range from Late Oligocene to present. This time span has been subdivided into 13 temporal intervals following the international chronostratigraphic chart of 2013^74^, from which numerical ages are taken.

### Speciation mode determination

Species level phylogenies are a key source of explanation for fossil record biogeographic patterns^75^,^76^. So as to determine the speciation events involving rhizomyines, the cladogram resulting from our analysis has been transformed into an area cladogram (Supplementary Figure 1). For this, the names of the terminal taxa and the ancestral nodes have been replaced with their geographic areas^75^. The geographical areas used in this work are specified in Supplementary Figure 1. A modified version of Fitch parsimony algorithm^77^ has been applied to determine geographic locations for the ancestral nodes. The nodes and terminal taxa are numbered as follows: the node at the root of the cladogram (n) was assigned a rank of one and each descendant node is given a rank of n + 1 (Supplementary Figure 1).

Speciation by vicariance has been recognized at nodes where the descendants occupy only a subset of the ancestral range, whereas speciation by dispersal has been identified at nodes where the descendants occupy geographic regions additional to or different from the ancestral range. Sympatric speciation has been identified at nodes where the descendants occupy the same biogeographic regions as the ancestral range^5^,^75^,^78^,^79^,^80^. The area-cladogram, in which the events of vicariance and geodispersal are marked, has been transformed into an area-stratocladogram (Fig. 1) so as to assess approximately the timing of each speciation event.

### Biodiversity rates

The combination of cladistic and biodiversity analyses highlights the phenomena of speciation, extinction, and diversity changes in a given group over time^5^,^81^. This allows establishing the timing of biodiversity crises and, thus, deducing possible causes.

Per-capita rates for speciation (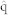), extinction (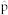), and diversity change (d) have been calculated (Supplementary Table 1) following the equations given by Foot^82^ according to which:
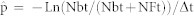

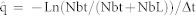

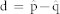
where Nbt indicates the number of species that cross both the upper and lower interval boundaries, NFt the number of species that originate within the interval and cross over the upper interval boundary and NbL the number of species that cross the lower interval boundary, but become extinct during the interval, and Δt the duration of the interval t_1_ − t_0_.

Rates of biodiversity change (R), speciation (S), and extinction (E) have been calculated (Supplementary Table 1) with the following equations^81^:
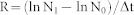

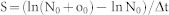

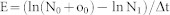
where N_0_ is the initial number of species in a clade at time t_0_, N_1_ the number of species in a clade at time t_1_, o_0_ the number of speciation events during the interval t_1_ − t_0_, and Δt the duration of the interval t_1_ − t_0_.

All rates were calculated for each temporal interval using the phylogenetically corrected species ranges obtained from the stratocladogram (Fig. 1). Values calculated from the first and last intervals have been excluded from the analysis to remove edge effects, following the criterion of Stigall^5^.

## Author Contributions

R.L.A. designed the study. R.L.A., F.K. and L.J.F. analyzed the data. R.L.A., F.K., L.J.F. and S.W. contributed to the discussion. R.L.A. wrote the paper.

## Supplementary Material

Supplementary Information Supplementary information

Supplementary Information Supplementary Table 1
