# Supplementary material for: Causal evidence between monsoon and evolution of rhizomyine rodents
Source: Sci Rep. 2015 Mar 11;5:9008. doi: 10.1038/srep09008 (PMC4355670; doi:10.1038/srep09008)

# SUPPORTING INFORMATION

# Causal evidence between monsoon and evolution of rhizomyine rodents

**Raquel López-Antoñanzas[1,3*], Fabien Knoll[1,2,3], Shiming Wan[4] & Lawrence J. Flynn[5]**

[1]School of Earth Sciences, University of Bristol, Bristol, United Kingdom, [2]School of Earth, Atmospheric & Environmental Sciences, University of Manchester, Manchester, United Kingdom, [3]Departamento de Paleobiología, Museo Nacional de Ciencias Naturales-CSIC, Madrid, Spain, [4]Key Laboratory of Marine Geology and Environment, Institute of Oceanology-Chinese Academy of Science, Qingdao, China, [5] Department of Human Evolutionary Biology, Harvard University, Cambridge, USA

**Supplementary Figure 1 | Paleobiogeographic patterns in extant and fossil rhizomyine rodents.** Each terminal taxon and every node is given a circled number. The area of occurrence (from 1 to 4) is numbered beside each, where 1=Asia, 2=Indian subcontinent, 3=N Africa, and 4=E Africa. Mapping the geographic states to the ancestral nodes using phases one and two of Fitch[77] optimization suggests that there have been several events of range expansion and contraction (denoted by D and V, respectively) during the evolutionary history of the group (see text).

**Supplementary Table 1 | Rate calculations. Instantaneous per-capita and deterministic rates calculated as described in the "Material and methods" section.** Raw observed turnover data include only the observed stratigraphic ranges of species, while phylogenetically constrained data incorporate ghost ranges. Rates are calculated from species turnover data to the left of the rate columns. Δt is the temporal duration of the interval determined from the 2013 chronostratigraphic chart[74]. NbL indicates the number of species that cross the lower interval boundary, but become extinct during the interval, NFt indicates the number of species that originate within the interval and traverse the upper interval boundary, Nbt indicates the number of species that cross both the upper and lower interval boundaries. $N_0$ indicates the number of species that are extant at the beginning of the interval of time $t_n$, Nf indicates the number of species that survive the end of $t_n$, # sp indicates the number of speciation events that occur during that interval, # ext indicates the number of species that experience extinction during it.

**Supplementary Figure 1**

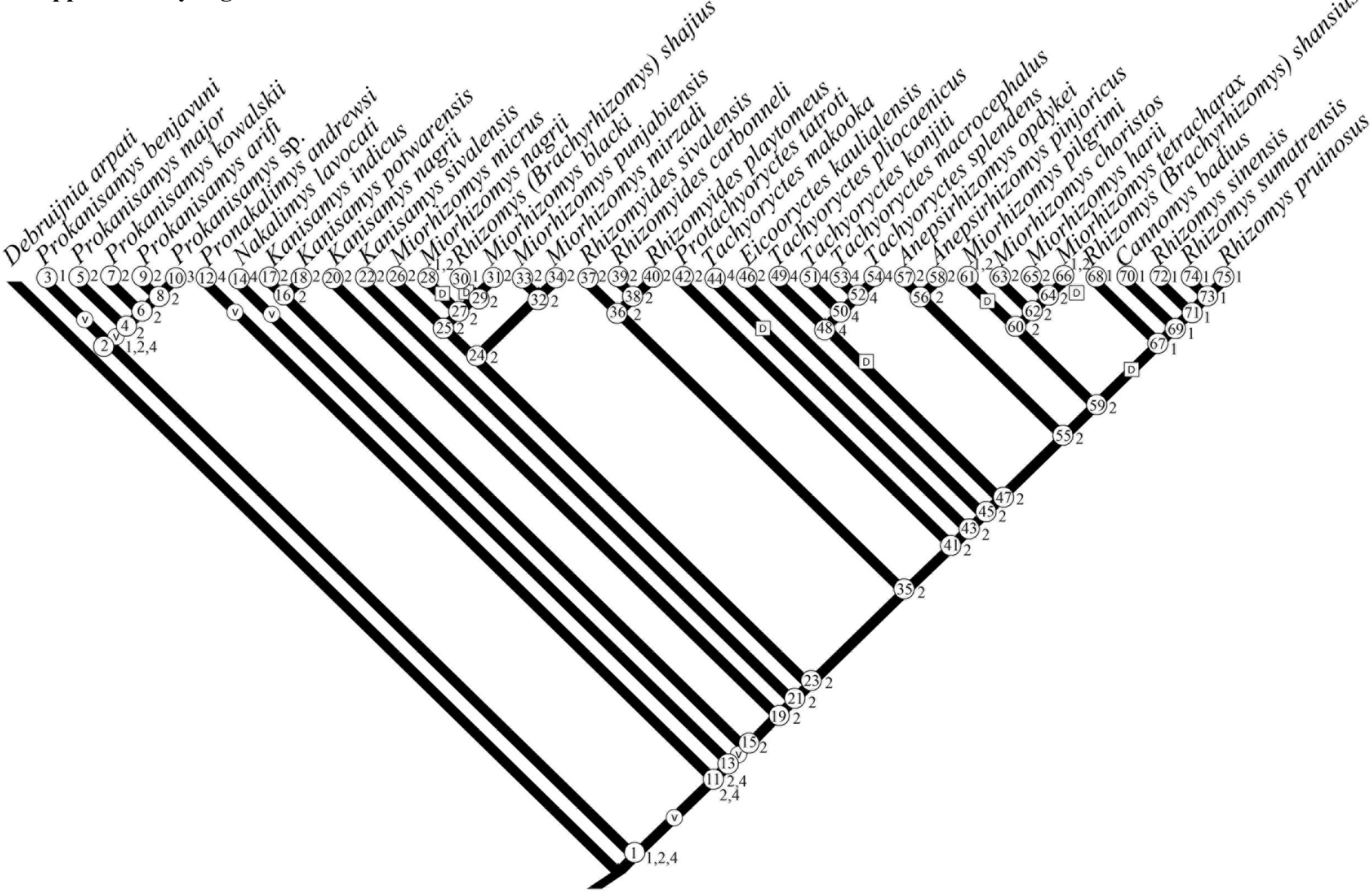

Supplement: Supplementary Information [file srep09008-s1.pdf]
